# The path forward for substance use disorder treatment using contingency management under sect. 1115 demonstration waivers

**DOI:** 10.1186/s13011-025-00666-6

**Published:** 2025-09-30

**Authors:** Vivian Kaufman, Devin C. Tomlinson, Lauren Hellman, Lewei A. Lin, Anne C. Fernandez, Lara N. Coughlin

**Affiliations:** 1 Addiction Center, Department of Psychiatry, University of Michigan, North Campus Research Complex, 2800 Plymouth Road, Building 16, Ann Arbor, MI USA; 2 Michigan Innovations in Addiction Care through Research and Education (MI-ACRE), Department of Psychiatry, University of Michigan, Ann Arbor, MI USA; 3 VA Center for Clinical Management Research (CCMR), VA Ann Arbor Healthcare System, Ann Arbor, MI USA

**Keywords:** Section 1115 Demonstration Waivers, Substance use disorders, Contingency management

## Abstract

**Introduction:**

Substance use disorders (SUDs) are a prevalent issue in the United States (U.S.) and there is a need for innovative treatments to address this public health issue. As of March 2025, there are seven states either approved or in the process of applying for Sect. 1115 Demonstration Waivers to implement pilot contingency management (CM) programs for SUD treatment. This manuscript qualitatively summarizes these Sect. 1115 Demonstration Waivers and the different aspects of each U.S. state's program.

**Methods:**

Data are from states Sect. 1115 Demonstration Waivers, collected through September 2024 and updated in March 2025. When possible the information has been verified with pilot program managers from each state, and pilot program managers have provided information when possible.

**Results:**

Eight states have applied for Sect. 1115 Demonstration Waivers to implement pilot CM programs. Five states have been approved (California, Washington, Montana, Hawaii, and Delaware), two are pending approval (Michigan, and Rhode Island) and one state’s CM application was denied (West Virginia). California is the only state to have confirmed implementing the Sect. 1115 Demonstration Waiver funding and has started to review evaluation data. The CM programs covered under Sect. 1115 Demonstration Waivers vary in substance targeted (e.g., stimulants, opioids), length of program (12–64 weeks), amount of incentives ($596–1092), and other characteristics (e.g. incentive delivery type, schedule of reinforcement).

**Conclusions:**

Section 1115 Demonstration Waivers addressing SUDs with CM are still new but with the increase in waivers approved, states who wish to apply and receive funding can learn from the approved waivers.

**Supplementary Information:**

The online version contains supplementary material available at 10.1186/s13011-025-00666-6.

## Introduction

### Increasing treatment of substance use disorders in the United States

In 2022, 70.3 (24.9%) million people ages 12 or older used illicit drugs in the past year [1]. Substance use disorders (SUDs) are prevalent and increasing with approximately one in four people (24.0%, 13.1 million people) classified as needing treatment for substance use in the past year receiving it [1]. There are many barriers to accessing substance use treatment including individual, social, and structural factors (e.g., fear, stigma, policies that promote enforcement over harm reduction, inaccessibility, inpatient care capacity, unaffordability) [2]. There are significant disparities in treatment access and utilization of SUDs among socioeconomically marginalized individuals [3, 4]. With the high prevalence of SUDs and low treatment uptake among socioeconomically marginalized populations, there is a need for highly effective treatments targeted at these individuals struggling with SUDs.

### Contingency management for substance use treatment

Empirically-supported SUD treatments vary depending on the category of SUD (e.g., opioids, alcohol, stimulants). For example, front-line treatments for alcohol use disorder include medications approved by the Food and Drug Administration (i.e., Naltrexone, Acamprosate, Disulfiram) and behavioral treatment [5]. Another class of SUDs is stimulant use disorder (StUD) which includes misuse of methamphetamine, cocaine, ecstasy, and prescription stimulants [6]. StUDs have high rates of overdoses; in 2017, one in five overdoses involved cocaine and one in seven involved a psychostimulant [7]. However, there are currently no FDA-approved medications for StUD, demonstrating a need for behavioral treatments [8, 9]. The greatest current empirical support is for the behavioral treatment termed Contingency Management (CM) [8, 9]. CM programs, (referred to as Recovery Incentives in California and Michigan programs), also known as motivational incentives and incentive-based treatments, provide immediate, monetary reinforcers for fulfilling a target behavior (e.g., substance use abstinence, treatment engagement) [10]. Studies have shown that use of CM is a highly effective treatment for reducing drug use in stimulants, as well as for other types of SUDs including with opioids, alcohol, tobacco, cannabis and benzodiazepines [11–15].

### Section 1115 demonstration waivers

Section 1115 Demonstrations (herein termed 1115 Waivers) funded through the Center for Medicare and Medicaid (CMS) are pathways for experimental, pilot, or demonstration projects that align with the goals of Medicaid [16]. The objective of 1115 Waivers is to allow states the flexibility to design and improve their programs [16]. CMS reviews each 1115 Waiver application to determine if they will have a positive impact on the Medicaid populations in that state and if they follow the requirements of these waivers. An important requirement of these waivers is that they must be “budget-neutral” to the Federal government and likely to furnish health for Medicaid beneficiaries in order to be approved [16]. 

### Current landscape of 1115 demonstrations for contingency management

In light of growing stimulant-involved overdose deaths and the recommendation for use of CM in the National Drug Control Strategy, several states have applied and received approval to add CM for SUDs to an existing 1115 Waiver. These specific programs covered under 1115 Waivers are typically termed Recovery Incentive or CM programs [7, 17]. These pilot programs vary in target behaviors (e.g., stimulants, opioids), length (e.g., 12–64 weeks), amount of motivational incentives available to patients (e.g., $596–1092), and other features (e.g., supplemental funding sources, eligibility criteria, schedule of reinforcement).

These waivers have been and can be used to implement new and innovative treatments for those affected by a variety of SUDs, but the common pathway for initial programs has been to focus on StUDs due to the aforementioned lack of effective treatment options for stimulants. The goal of the present narrative review is to provide an overview of 1115 Waivers on CM programs to better understand the scope of current waivers and implementations in each state. The goal of the narrative review is to help inform states wishing to apply and implement a CM program through 1115 waivers.

## Methods

This review summarizes each state's CM program that is under an 1115 Waiver. To begin the narrative review, a search was done on the CMS 1115 waiver list website [18]. Further research was performed with states that had public records of an application for an 1115 Waiver that included CM. To compile information on the current 1115 Waivers pulled from the CMS website, we researched publicly available resources and also contacted programs individually to further understand the context of current programs. In this report, we focus on the similarities, and differences across states to help set the stage for future comparisons of the CM programs. Policy details reviewed are as of March 2025. Policies and 1115 Waivers, like those described below, can change rapidly in states, and the number of states that have approved/pending/implemented recovery incentive programs through Sect. 1115 Demonstration waiver funding may have already evolved following the submission of this publication.

Policy data reviewed in this paper was collected over 4 months, from July 2024 to September 2024, and updated in March 2025 through publicly available state-enacted rules and regulations (see Supplemental Table 1) and informally validated through state public health officials via email and face-to-face correspondence. Data was collected to provide a real-time view of the current status of Sect. 1115 Waivers on CM to better understand the scope of these waivers and their implementation in each state.

## Results

**Fig. 1:**
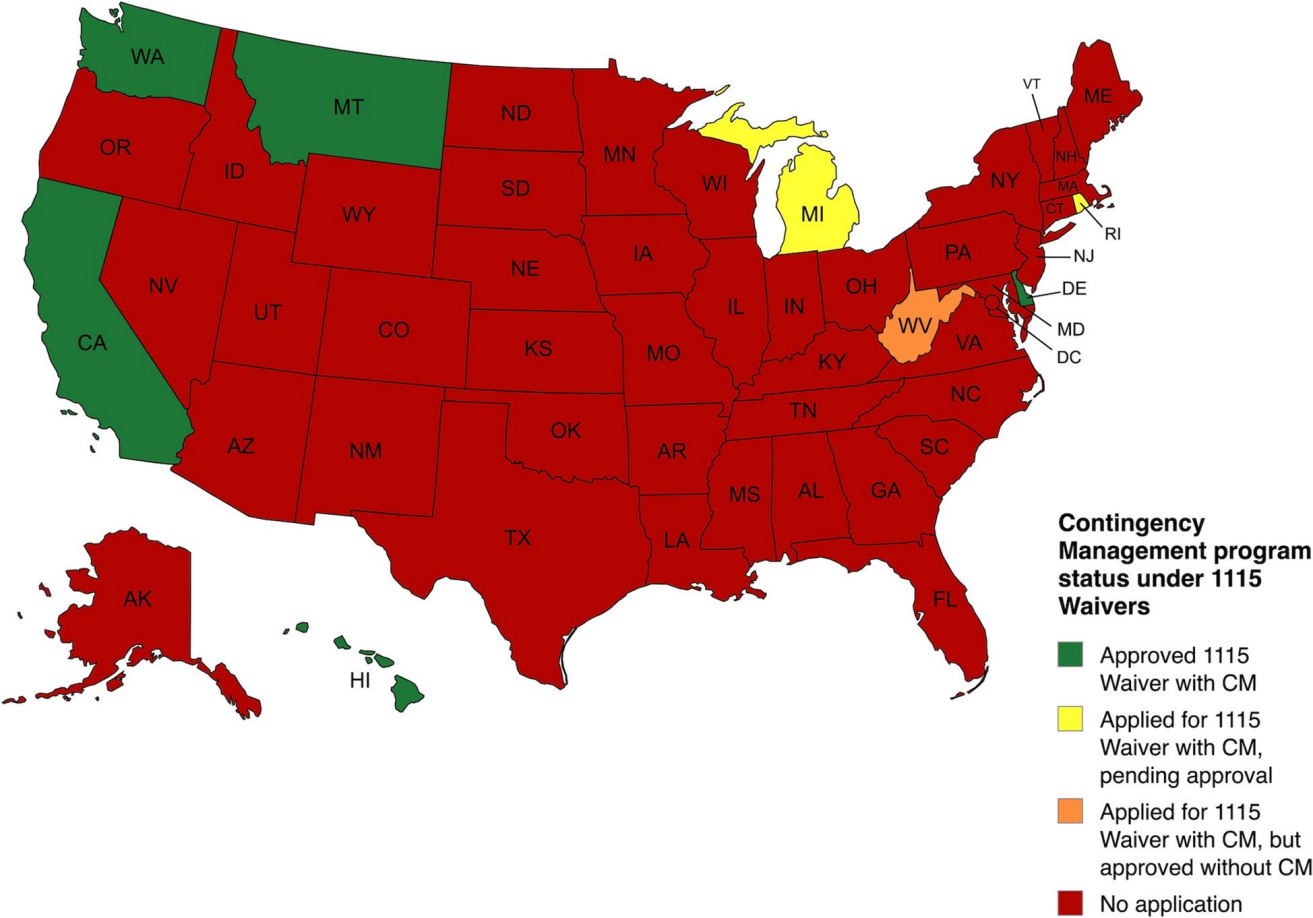
State of 1115 Waivers with CM programs as of March 31, 2025

As of March 31, 2025, eight states have applied for an 1115 Waiver that included CM programs for SUD. Five states have been approved (California, Washington, Montana, Hawaii, and Delaware), two are pending approval (Michigan and Rhode Island) and one state’s application was denied (West Virginia). Of the states approved, California is the only state confirmed to begin implementing the CM benefit (services that the health plan covers) from Sect. 1115 Waiver funding. See Fig. 1 for a map of the current landscape in the United States and Tables 1 and 2 for a comparison of each state's 1115 Waiver and specific program aspects. Importantly, the programs covered in this paper are based on 1115 Waivers, thus, all programs are limited to Medicaid beneficiaries.

**Table 1:** Contingency Management Programs Applied for in 1115 Waivers by State

State	Section 1115 Demonstration Waiver Name	Contingency Management Program Name	Date Applied For Contingency Management Program In The 1115 Waiver	Date Approved	1115 Waiver Expiration	Population Covered Under 1115 Waiver	Program Length	Biochemical Verification Method	Maximum Incentive Approved For ($)	Participant Visit Frequency
California	California Advancing and Innovating Medi-Cal (CalAIM) Waiver	Recovery Incentives Program	6/30/2021	7/1/2022	12/31/2026	Beneficiary with a substance use disorder	24 weeks	POC urine drug testing	599	Weeks 1–12: 2x/week Weeks 13–24: 1x/week
Washington	Washington Medicaid Transformation Project	Contingency Management for SUD Treatment	7/1/2022	6/30/2023	6/30/2028	Beneficiary with a substance use disorder	24 weeks^5^	POC urine drug or oral test^5^	1092^5^	Weeks 1—12: 2x/week Weeks 13–24: 1x/week^5^
Montana	The Montana Healing and Ending Addiction through Recovery and Treatment (HEART) Waiver	Contingency Management program	10/1/2021^1^	2/26/2024	7/30/2027	Beneficiary with stimulant use disorders	12 weeks^5^	POC urine drug testing^5^	596^5^	Weeks 1—12: 2x/week^5^
Delaware	Delaware Diamond State Health Plan (DSHP) Sect. 1115 Demonstration	Delaware's Contingency Management Services for Certain Beneficiaries with a Stimulant Use Disorder and/or Opioid Use Disorder	12/30/2022	5/17/2024	12/31/2028	Beneficiary with stimulant use disorder or adults that are pregnant or up to 12 months postpartum diagnosed with an opioid use disorder^2^	12 week program and 64 weeks program^3, 5^	POC urine drug testing^5^	750^4^^, 5^	N/A
Hawaii	Hawaii QUEST Integration	N/A	Late 2023/Early 2024	1/8/2025	12/31/2029	Beneficiaries with a stimulant use disorders and/or opioid use disorder	24 weeks^5^	N/A	N/A	N/A
Michigan	Michigan 1115 Behavioral Health Demonstration (formerly Pathways to Integration)	Recovery Incentives (RI) Pilot	4/1/2024	N/A	N/A	As proposed in 1115 request: Beneficiary with a stimulant use disorders, opioid use disorder, or both^5^	24 weeks^5^	POC urine drug testing^5^	599^5^	Weeks 1—12: 2x/week Weeks 13–24: 1x/week^5^
Rhode Island	Rhode Island Comprehensive Demonstration	Contingency Management Pilot Program	5/23/2024	N/A	N/A	As proposed in 1115 request: Beneficiary with alcohol and/or substance use disorder^5^	N/A	POC urine drug testing^5^	599^5^	N/A
West Virginia	West Virginia Creating a Continuum of Care for Medicaid Enrollees with Substance Use Disorders	N/A	5/27/2022	N/A	N/A	N/A	N/A	N/A	N/A	N/A

^1^Montana first applied on 10/1/2021 but the CM component was not approved. Montana reapplied on 2/15/2024 for an amendment to add the CM program

^2^ Delaware’s 1115 Waiver approved two groups for CM 1. beneficiary with a stimulant use disorder and 2. adult beneficiaries that are pregnant or up to 12 months postpartum diagnosed with an opioid use disorder

^3^ Beneficiaries with a stimulant use disorder are approved for a 12-week program, and adult beneficiaries that are pregnant or up to 12 months postpartum diagnosed with an opioid use disorder are approved for a 64-week program

^4^ Delaware originally applied for a maximum incentive amount of $599 but later with the approval of CMS the incentive amount increased to $750 a year for both groups

^5^ This information is currently based on state program information for states not currently using 1115 Waiver funds, this information is subject to change prior to implementing the 1115 Waiver demonstration

Abbreviations: POC = Point-of-care

**Table 2:** Characteristics of Contingency Management Programs under 1115 Waiver by State

State	Date implemented/planned implementation	Population of focus in the pilot program	Training and technical assistance (TTA) provider	Incentive Manager platform	Incentive type
California	3/1/2023	Beneficiary with stimulant use disorder	UCLA Training and Implementation team	Q2i	E-Gift Cards
Washington	Early 2025^2^	Beneficiary with stimulant use disorder and/or opioid use disorder	Washington State University (WSU), Promoting Research Initiatives in Substance Use and Mental Health (PRISM)^2^	REDCap^2^	E-Gift Cards^2^
Montana	Early 2025^2^	Beneficiary with stimulant use disorders^2^	UCLA Department of Psychiatry and Vermont Center on Behavior and Health^2^	REDCap^2^	E-Gift Cards^2^
Delaware	N/A	Beneficiary with stimulant use disorder or adults that are pregnant or up to 12 months postpartum diagnosed with an opioid use disorder^1,2^	N/A	N/A	N/A
Hawaii	N/A	Beneficiary with a stimulant use disorder, and/or opioid use disorder^2^	N/A	N/A	N/A
Michigan	N/A	As proposed in 1115 Waiver request: Beneficiary with stimulant use disorder, opioid use disorder, or both^2^	Altarum^2^	CHESS Health^2^	Reloadable debit cards^2^
Rhode Island	N/A	As proposed in 1115 Waiver request: Beneficiary with alcohol and/or substance use disorder^2^	DynamiCare^2^	DynamiCare^2^	N/A
West Virginia	N/A	N/A	N/A	N/A	N/A

^1^Delaware’s 1115 Waiver approved two groups for CM 1. beneficiary with a stimulant use disorder and 2. adult beneficiaries that are pregnant or up to 12 months postpartum diagnosed with an opioid use disorder

^2^This information is currently based on state program information for states not currently using 1115 Waiver funds, this information is subject to change prior to implementing the 1115 Waiver demonstration

Abbreviation: N/A = Not Available

### California

The first state to implement CM programs through an 1115 Waiver was California in 2023. Through this waiver titled “California Advancing and Innovating Medi-Cal (CalAIM)”, the California Department of Health Care Services (DHCS) is approved to use the CM benefit for qualifying members with any SUD [19]. Currently, the DHCS is utilizing the CM benefit for the Recovery Incentives Program [20]. The Recovery Incentives Program was approved for the pilot period of July 1, 2022 through December 31, 2026 [19], and the program began implementation in March 2023 (personal communication with a member of California’s DHCS, July 10—September 11, 2024).

#### Eligibility

While the DHCS approved for use of CM for qualifying members with any SUD, the Recovery Incentives Program is specifically for qualifying members with a StUD (personal communication with a member of California’s DHCS, July 10—September 11, 2024). To be eligible for the Recovery Incentives Program, beneficiaries must be enrolled in a comprehensive treatment program, and be assessed to have a StUD [19]. Patients must reside in one of the 19 participating counties to receive this benefit [19]. Beneficiaries, including adolescents, who meet eligibility criteria will be able to participate in the Recovery Incentives Program [19]. There is no age restriction for CM services. Minors under age 12 are eligible to participate with parental consent [19]. Minors ages 12–20 who participate in the Minor Consent program (i.e., minors who wish to receive confidential care under California Code of Regulations, Title 22, Sect. 51,473.2 [21]) do not need parental consent to participate in the program [19].

#### Program structure

The program is a structured 24-week outpatient program [19]. Weeks 1–12 are the escalation/reset/recovery period with in-person visits twice a week, and weeks 13–24 are the maintenance period with in-person visits once a week [19]. After the 24 weeks, participants are enrolled in 6 or more months of aftercare and treatment services to support ongoing recovery [19]. Participants earn incentives that increase over weeks 1–12 with negative point-of-care (POC) urine tests [19]. During weeks 1–12 participants can earn a max of $438, with the incentive starting at $10 and increasing by $1.50 with each negative test week [19]. During weeks 13–24 the maximum amount a participant can earn is $161, with weeks 13–18 negative tests equaling $15 per week, weeks 19–23 negative tests equaling $10 per week, and week 24’s final negative test equaling $21 [19]. Over the course of the program participants can earn up to $599 [19]. If a participant submits a positive test or has an unexcused absence, a “reset” will occur and the next negative test incentive will be at the original value [19]. If a participant submits two consecutive negative tests following this reset, a “recovery” of their pre-reset value will occur [19]. There is ambiguity surrounding whether the incentives earned are considered income by the Internal Revenue Service (IRS). According to the waiver, the IRS is expected to make a decision on whether incentives earned under CM are considered income or are exempt due to being part of medical treatment [19].

#### Training and technical assistance (TTA)

Throughout the implementation and course of the pilot, TTA is being run by the University of California Los Angeles (UCLA) Training and Implementation team [22]. All training sessions were conducted virtually and synchronously [22]. There were required training and steps for a county to prepare for implementation which included CM Overview Training, Specific CM Protocol Implementation Training, Understanding of the OIG’s Final Rule and Operational Guidelines Training, and a Readiness Review [22]. Technical assistance was provided throughout the pilot period both virtually and on-site, as needed [22].

#### Incentive delivery

The results of the POC urine tests are immediately delivered by the CM coordinator to the participating beneficiary and the rewards are disbursed immediately once results are received through the the Incentive Manager platform Q2i [23] (personal communication with a member of California’s DHCS, July 10—September 11, 2024). Participants can choose from a range of gift cards from retail outlets or can redeem the balance. Restrictions are placed on the money, not allowing purchases of cannabis, tobacco, alcohol, or lottery tickets [19].

#### Funding

Apart from 1115 Waiver funding, the program is supported through a combination of funding sources. This includes allocations from Behavioral Health Quality Improvement Program funding and Substance Abuse and Mental Health Services Administration (SAMHSA) block grant funding, totaling $5,640,000 to support start-up program costs, which were provided to opt-in counties in May 2022 [24, 25] (personal communication with a member of California’s DHCS, July 10 - September 11, 2024). These funds were for start-up activities during the fiscal year 2021-2022. DHCS also allocated an additional 2 million dollars in SAMHSA block grant funding to be used by September 30, 2023 [24].

Through August 15, 2024, the program was funded by state General Funds and federal Medicaid matching funds [24, 25] (personal communication with a member of California’s DHCS, July 10 - September 11, 2024). State funds were made available by enhanced federal funding provided through the CMS-approved Home and Community-Based Services (HCBS) Spending Plan [24, 25] (personal communication with a member of California’s DHCS, July 10 - September 11, 2024). Since August 15, 2024, after the close of the HCBS Spending Plan, counties are now responsible for supporting the non-federal share of costs, including CM services, administrative funding, and incentive costs [24, 25] (personal communication with a member of California’s DHCS, July 10 - September 11, 2024). The Incentive Manager software will still be funded by DHCS, as will TTA and evaluation contracts using state General Funds and federal funds. More information about DHCS’ fiscal year 2023-24 and 2024-25 budgets can be found here [24, 25] (personal communication with a member of California’s DHCS, July 10- September 11, 2024).

#### Implementation

There are currently 19 active counties delivering CM services and four others preparing to begin (personal communication with a member of California’s DHCS, July 10 - September 11, 2024). The Recovery Incentives Program has recently been approved for expansion and an Implementation Plan has been sent out to the rest of the eligible DMC-ODS counties for DHCS approval (personal communication with a member of California’s DHCS, July 10 - September 11, 2024). DHCS is actively working with these new counties and UCLA, the program TTA contractor, to support program implementation [20, 26] (personal communication with a member of California’s DHCS, July 10 - September 11, 2024).

### Washington

Washington was the next state to be approved for a CM pilot program through 1115 Waiver and closely followed California's program and structure. The 1115 Waiver titled “Washington Medicaid Transformation Project 2.0 (MTP 2.0)” was approved on June 30, 2023 for a period starting July 1, 2023 through June 30, 2028 [27, 28]. Washington’s waiver was written to cover three substances (stimulants, opioids and alcohol) but the Washington State Health Care Authority (HCA) approved only stimulants and opioids and is starting the program with a focus on StUDs, similar to California (personal communication with a representative from the Washington State HCA, September 26, 2024; confirmed March 27, 2025) [27]. The 1115 Waiver will cover the cost of incentives and POC tests but does not cover clinician time.

#### Eligibility

The eligibility requirements are the same as California's program, detailed above [27]. As mentioned above, beneficiaries must be diagnosed with a StUD (personal communication with a representative from the Washington State HCA, September 26, 2024; confirmed March 27, 2025). The only difference in eligibility requirements is that Washington residents qualify for the program no matter where they live and are not restricted by the county they live in (personal communication with a representative from the Washington State HCA August 8, 2024; confirmed March 27, 2025).

#### Program structure

The MTP 2.0 is approved to follow the same 24-week structure as California's program, but the maximum incentives a participant can earn is double the amount at $1,092 [27]. According to the waiver, during weeks 1-12 weeks participants are asked to visit the treatment setting a minimum of two treatment visits per week separated by at least 72 hours [27]. During weeks 1-12 participants can earn a maximum of $528, with the incentive starting at $10 and increasing by $2 with each negative test week [27]. During weeks 13-24 participants are asked to visit the treatment facility for testing at least once a week [27]. The maximum a participant can earn during weeks 13-24 is $564 with the incentive for each visit increasing by $2 with each negative test week [27]. The HCA is currently approving the maximum incentive of $599, but with the goal to reach $1092 the amount approved under the 1115 Waiver (personal communication with a representative from the Washington State HCA, September 26, 2024; confirmed March 27, 2025).

#### TTA

Throughout the implementation and course of the pilot, TTA is being run by Washington State University (WSU), Promoting Research Initiative In Substance Use and Mental Health (PRISM) [29].

#### Incentive delivery

Throughout the implementation and course of the pilot, the the Incentive Manager platform being used is REDCap and the same delivery methods and restrictions are placed on the rewards as California’s program (personal communication with a representative from the Washington State HCA August 8, 2024; confirmed March 27, 2025).

#### Funding

Besides 1115 Waiver funding, this program is funded through a Biennial operating budget, $500,000 General Fund State- Fiscal Year 2024 and $500,000 General Fund State- Fiscal Year 2025 (personal communication with a representative from the Washington State HCA August 8, 2024; confirmed March 27, 2025). There was additional funding for staff and training through the State Opioid Response III (SOR) grant but is no longer being used (personal communication with a representative from the Washington State HCA August 8, 2024; confirmed March 27, 2025).

#### Implementation

Washington has yet to implement the expansion of the program with 1115 Waiver funding but is planning to have sites running in early 2025 (personal communication with a representative from the Washington State HCA, September 26, 2024; confirmed March 27, 2025). The program had 9 clinics operating before receiving 1115 Waiver approval and currently has 11 clinics providing CM and will add 10 sites every year under the 1115 Waiver (personal communication with a representative from the Washington State HCA, September 26, 2024; confirmed March 27, 2025).

### Montana

Montana was the third state to be federally approved for their pilot program under the 1115 Waiver “Montana Healing and Ending Addiction through Recovery and Treatment (HEART)”. Montana’s 1115 Waiver only includes covering services for individuals with StUD, which differs from California and Washington [30]. Montana originally applied for the HEART waiver on October 1, 2021, seeking approval for 1115 Waiver funds to cover CM [30] as the HEART waiver was approved without CM [30]. On February 15, 2024 Montana sought an amendment to add more services to the HEART waiver, including CM, and was approved on February 26, 2024 [30, 31]. The HEART demonstration waiver was approved for the period of October 1, 2024 through June 30, 2027 [30].

The Treatment of Users of Stimulants (TRUST) pilot program, which started before 1115 Waiver approval, included CM for people with StUDs [32]. The Montana State government decided to separately offer CM from the TRUST program and once the 1115 Waiver is enacted, CM costs will be reimbursable through Medicaid alone (personal email communication with a member of Montana DPHHS [Department of Public Health & Human Services], July 31, 2024; confirmed March 12, 2025).

#### Eligibility

The eligibility requirements are closely aligned with California’s; beneficiaries must be assessed and diagnosed with a StUD. The major difference is that Montana requires patients receiving the CM benefit to be 18 years or older [32].

#### Program structure

The structure of Montana's CM is a 12-week intensive intervention followed by up to 9 months of aftercare and continued recovery and stabilization. During the 12 weeks, participants are asked to visit the treatment facility at least twice per week separated by 72 hours [30]. The initial reward for a negative test is $12 and can increase by $2 every other visit of negative tests [30].The maximum annual amount a participating beneficiary can receive is $596 [30]. This program is half the length of California’s but with a similar incentive amount. The number was chosen so it would fall under the “general welfare exclusion” federal tax exemption [30]. This ensured it would be excluded from participating beneficiaries’ modified adjusted gross income (MAGI)-based eligibility determinations, non-MAGI-based eligibility determinations, and share of cost determinations so the incentive reward would not be a determining factor in those beneficiaries’ eligibility for Montana Medicaid [30].

#### TTA

In the pilot program, the TTA is being run by the UCLA Department of Psychiatry and Vermont Center on Behavior and Health [32].

#### Incentive delivery

Montana is working with WSU using their RedCap as the incentive tracker in the pilot program (personal email communication with a member of Montana DPHHS, July 31, 2024; confirmed March 12, 2025).

#### Funding

The majority of the funding will be through 1115 Waiver funding, but currently CM is funded primarily through a federal State Opioid Response (SOR) grant from the SAMHSA [32]. When the CM program is a payable service, SOR funding will continue to fund people who are not Medicaid eligible or are under-insured or have no insurance so they can still receive CM services (personal email communication with a member of Montana DPHHS, July 31, 2024; confirmed March 12, 2025).

#### Implementation

Montana has yet to enact the CM benefit under the 1115 Waiver (personal email communication with a member of Montana DPHHS, July 31, 2024; confirmed March 12, 2025). Montana plans to roll out the benefit early in 2025 (personal email communication with a member of Montana DPHHS, July 31, 2024; confirmed March 12, 2025).

### Delaware

Delaware's CM program titled “Services for Certain Beneficiaries with a Stimulant Use Disorder and/or Opioid Use Disorder” under 1115 Waiver the “Delaware Diamond State Health Plan (DSHP)” was approved on May 17, 2024 and is effective until December 31, 2028 [33]. The project team contacted the Division of Medicaid and Medical Assistance in Delaware but limited information was provided.

#### Eligibility

The program has similar eligibility requirements to California’s requirements but requires that patients receiving the CM benefit be age 18 years or older [33]. Delaware has a two-part program, the first part of which is similar to California's program covering individuals with a StUD (group CM-StUD). The second part of the program is specifically for people who are pregnant or up to 12 months postpartum diagnosed with an opioid use disorder (OUD; group CM-PPPOUD) [33].

#### Program structure

Group CM-StUD has the same program structure as California, lasting 24 weeks. Group CM-PPPOUD patients are eligible for a 64-week program [33]. The full details of the program (e.g., reinforcement schedule) are still being finalized (personal communication with a member of Delaware's Division of Medicaid & Medical Assistance [DMMA], July 9, 2024). In the original application, the maximum a participant could earn was $599 in incentives each year for both groups [33]. The Division of Medicaid & Medical Assistance selected $599 as the maximum incentive amount because it is the most an individual can receive without paying taxes on these funds [33]. After discussing with CMS, the incentive amount was increased to $750 a year for both groups due to the evidence that shows that amount being more effective (personal communication with a member of Delaware's DMMA, March 13, 2025). The Delaware team worked with CMS and the Treasury Department to get written approval for the increase and was assured that the incentives would not be counted as income as this may be a barrier to participation (personal communication with a member of Delaware's DMMA, March 13, 2025).

#### Program details

Delaware is still working to choose an incentive management platform (personal communication with a member of Delaware's DMMA, March 13, 2025). Depending on the incentive management platform chosen, the mode of incentives could be either e-gift cards or debit cards (personal communication with a member of Delaware's DMMA, March 13, 2025). Attachment K which will include more comprehensive details is currently waiting for approval from CMS and is not available at this time (personal communication with a member of Delaware's DMMA, April 15, 2025). Other information on the details of the program was not available at the time of this publication.

### Hawaii

Hawaii was approved for a pilot CM program under their 1115 Waiver “Hawaii Quest Integration” which was originally approved in 1993[34]. Hawaii applied in late 2023/early 2024 for an extension of the Hawaii Quest Integration and included contingency management in their application [35]. This extension and the program was approved on January 8, 2025, through the period of December 31, 2029.[34].

#### Eligibility

The program has similar eligibility requirements to California’s requirements, but Hawaii’s CM program in the 1115 Waiver specifically covers beneficiaries who are 18 years or older diagnosed with a StUD disorder and/or OUD.

#### Program structure

The CM benefit follows the same 24-week structure as California's program [34]. The maximum incentive amount a participant can earn and the full incentive were not specified in the waiver as Attachment K is yet to be updated [34].

#### Program details

Other information on details of the program was not available at the time of this publication.

### Michigan

Michigan applied for an extension on their 1115 Waiver “Michigan 1115 Behavioral Health Demonstration” (formerly Pathways to Integration), which was first approved in 2019 [36]. The state added The Recovery Incentives (RI) Pilot for the demonstration period of October 1, 2024 through September 30, 2026 [36]. As the recovery incentive through the waiver is still pending approval through CMS, many of the specifics of the program are still not finalized and subject to change [36]. Michigan Department of Health and Human Services (MDHHS) intends the pilot to run for two years, with the pilot period starting January 1, 2025 through December 31, 2026 [37].

#### Eligibility

Michigan’s program specifically covers CM for individuals living with StUD, OUD, or both [36]. Patients must be enrolled in Healthy Michigan Plan (HMP) or Medicaid and be enrolled in a Prepaid Inpatient Health Plan (PIHP) that elects and is approved by Michigan Department of Health & Human Services to provide the CM benefit [38]. Michigan currently has six PIHP regions approved to offer CM [36] (personal communication with MDHHS RI Pilot Coordinator, August 6, 2024; confirmed March 18, 2025). Pregnant individuals are eligible to participate in this program, and there are no age restrictions (personal communication with MDHHS RI Pilot Coordinator, September 12, 2024; confirmed March 18, 2025).

#### Program structure

The RI pilot follows the same 24-week structure as California's program, with 6 months of aftercare and recovery support services. The maximum incentive amount a participant can earn is $599 [36]. The incentive reinforcement schedule is not yet in the 1115 Waiver; however, it is outlined in the Provider Handbook under section 5.5, following a similar incentive schedule as California [37].

#### TTA

Altarum Institute will provide TTA throughout the implementation of the pilot [39] (personal communication with MDHHS RI Pilot Coordinator, August 6, 2024; confirmed March 18, 2025).

#### Incentive delivery

The pilot program will be using CHESS Health (www.chess.health) as the Incentive Manager platform [40].

#### Funding

Until the Waiver is approved, the pilot will be using Opioid Settlement Funds through the Michigan Opioid Healing and Recovery Fund (personal communication with MDHHS RI Pilot Coordinator, August 6, 2024; confirmed March 18, 2025). Pending approval, Federal and State Medicaid funds and Opioid Settlement Funds will be used to support the program (personal communication with MDHHS RI Pilot Coordinator, August 6, 2024; confirmed March 18, 2025).

#### Implementation

The pilot program launched services in April 2025 [38] (personal communication with MDHHS RI Pilot Coordinator, April 17, 2025).

### Rhode Island

On May 23, 2024, Rhode Island added to its extension request for its 1115 Waiver titled the “Rhode Island Comprehensive Demonstration” [41]. This addendum would add CM services. It is still pending approval by CMS [41] .

#### Eligibility

If approved, beneficiaries could be eligible for participation if they have an alcohol and/or SUD for which the CM benefit is medically appropriate and are actively enrolled in a comprehensive treatment program [41] .

#### Program structure

No public information regarding program structure was available at the time of this writing.

#### TTA

In the current version of the program, the Department of Behavioral Healthcare, Developmental Disabilities & Hospitals (BHDDH) is working with DynamiCare to ensure proper CM training and evaluation [42].

#### Incentive delivery

In the current version of the program, which is not funded by Medicaid, Rhode Island is using DynamiCare as their Incentive Manager platform [42].

#### Funding

Rhode Island is currently funding this work with the state’s Opioid Settlement Funds [42].

#### Implementation

Rhode Island’s 1115 Waiver has yet to be approved. However, there is an operational CM program using other funding sources [42].

### West Virginia

West Virginia applied for an 1115 Waiver titled “West Virginia Creating a Continuum of Care for Medicaid Enrollees with Substance Use Disorders” on May 27, 2022, and the waiver included a plan for a CM pilot program [43]. The passage of the waiver was delayed until January 17, 2025 and in the approved waiver, now titled “Evolving West Virginia Medicaid’s Behavioral Health Continuum of Care,” the CM program is not included [44]. In a document from December 11, 2024, CMS commented that the CM program would not be approved since “West Virginia’s demonstration does not generate budget neutrality savings, CMS cannot approve the state’s contingency management proposal” [45]. Correspondence with the program contact indicated there are no plans to go forward for a new approval (personal communication with West Virginia 1115 Waiver Program Manager, July 9, 2024). Even though West Virginia was not approved for CM under an 1115 Waiver, there are still organizations completing pilot CM programs, including through DynamiCare via managed care organizations [46–48].

## Discussion

This report summarizes a timestamp of the CM landscape in the U.S as of March 2025 with respect to 1115 Waivers. In our review, we identified five states with approved and two states with pending 1115 Waivers for CM programs from 2022 to 2025. Presently, all states with CM under an 1115 waiver include people with StUD in their CM programs, but several stateshave the latitude to implement CM programs for other SUDs. This is extremely promising, given empirical support for this type of treatment to improve outcomes across SUDs [49]. Other key differences across state programs include the incentive amounts ($596-$1,092), length of program (12–64 weeks), substance targeted (e.g., stimulants, opioids), alternative funding sources (e.g., SAMHSA, State Opioid Funds), Incentive Manager platforms (Q2i, REDCap, CHESS Health), who is doing the TTA (UCLA Training and Implementation team, WSU, PRISM, Altarum Institute), and other characteristics (e.g., incentive delivery type, schedule of reinforcement).

CM is gaining greater attention in the substance use treatment landscape [50, 51]. In parallel to the number of states applying for 1115 Waivers, SAMHSA recently published a report in January 2025 with guidelines for CM programs funded by SAMHSA [50]. In this report, recommendations for CM programs and guidelines for programs that apply for SAMHSA funding are provided [50]. SAMHSA officially changed its policy regarding CM activities in treating SUDs to provide incentive values of up to $750 per patient per year; this change better aligns incentive values with scientific evidence for CM [50]. Further, the report provides further evidence for the treatment community supporting implementation of evidence-based CM care for individuals with StUD and other SUDs [50]. Although a formal evaluation is outside the scope of this current report, there are early reports coming out of Californian Recovery Incentives demonstrations, and we anticipate additional reports will be forthcoming as programs have more time in the field [52–54].

Five states (CA, WA, MT, DE, HI) have been approved through the Medicaid demonstration for CM programs, with only one state confirmed currently (CA) implementing CM programs. Research studies across the country have shown CM to be an effective treatment for a variety of SUDs, including stimulants, opioid, tobacco, alcohol, cannabis and multiple substances [55, 56]. Both California and Washington include any SUD but are currently focusing on StUDs for their first pilot program. These two states, which already have approval, are able to expand their programs to include tobacco or alcohol use disorders, while other states like Montana, Delaware, Hawaii and Michigan would need to reapply if they were to expand outside of the approved substances.

The results of this report support states applying for 1115 Waivers for CM for SUDs, and that states applying can learn from the states with current programs. States wishing to apply and implement may benefit from consulting with states with successful applications and implementations to learn the processes and try to anticipate CMS priorities in this space. The timing from application to approval to implementation has varied across states. The length from application to approval was one year for both California and Washington and one year and five months for Delaware [19, 27, 33]. Michigan and Rhode Island are still waiting for approval twelve and ten months later, respectively [36, 41]. California’s 1115 waiver was approved on July 1, 2022 and began implementation in March 2023, nine months after original approval. Other states with approval are still working toward implementation [27, 30, 33, 34]. A more concrete timeline for implementation is not clear, as California is the only state to have started at the date of this report. Furthermore, all states except Montana have incorporated the CM program into existing 1115 waivers. It may be the case that embedding CM within other SUD care delivered through 1115 Waivers helps offset requirements related to cost neutrality. Moreover, states may consider other funding sources to sustain delivery of CM for SUD.

Another future direction for these and other pilot programs could be to implement digital CM or telehealth-delivered CM programs. Digital CM programs have been shown to be effective in treating SUDs [14]. Digital CM programs can help expand incentive-based treatment reach and engagement by allowing people in rural areas or areas with less health centers to access treatment, and any patients who would benefit from greater treatment accessibility, potentially addressing SUD treatment shortages [14]. Similarly, telehealth CM, wherein CM visits are still synchronous with a clinician but via telehealth platforms, may help to alleviate patient-level barriers (e.g., transportation) to CM engagement and enhance accessibility for the general patient population. Yet, states with approved 1115 Waivers and publicly available CM protocols have explicitly stated in-person CM delivery in their 1115 Waivers. In the future, by expanding use of 1115 Waivers to digital and/or telehealth CM interventions and collecting samples via remote methods (e.g., transmitting test results digitally or via video), care could potentially be expanded to cover more individuals and reach people who may face barriers to regular access to in-person care (e.g., childcare responsibilities, lack of reliable transportation, work scheduling limitations, living far from a clinic).

The present study is not without limitations. First, the report began by searching for publicly available applications for 1115 Waivers for CM. Therefore, we may have missed states at different steps in the application process. Second, this report is based on available published and information provided via outreach to state officials, however, it is not exhaustive and some information may be out of date by the time of this publication. Third, information on eligibility criteria might not be exhaustive, as some special populations may be eligible for the pilots but are not explicitly noted in the waivers, and/or confirmed by state contacts. Fourth, a limitation that states who are interested in applying for a CM program under an 1115 Waiver might take note of is that a state without budget neutrality savings might face barriers to approval of their program, as was seen with West Virginia’s Waiver application.

## Conclusion

This brief report outlines the landscape of CM programs through 1115 Waiver. It outlines the specifics of each program, highlighting key similarities and differences. We are at the forefront of state-level implementations of CM programs, and there is far more work to be done as CM programs spread across the country (see Fig. 1). This report adds context to both the CM program evaluation landscape and for states looking to apply for an 1115 Waiver to implement a CM program. This subset of information current as of March 2025 may provide models and lessons learned for the other states that are in the midst of planning their CM approach, as well as those who may consider CM in the future.

## Supplementary Information

Supplementary material 1.

## Data Availability

No datasets were generated or analysed during the current study.

## Abbreviations

BHDDH: The Department of Behavioral Healthcare, Developmental Disabilities & Hospitals
CM: Contingency management
CMS: Center for Medicare and Medicaid
CalAIM: California Advancing and Innovating Medi-Cal
DHCS: The California Department of Health Care Services
DMMA: Division of Medicaid & Medical Assistance
DPHHS: Department of Public Health & Human Services
DSHP: Delaware Diamond State Health Plan
HCA: The Washington State Health Care Authority
HCBS: Home and Community-Based Services
HEART: Montana Healing and Ending Addiction through Recovery and Treatment
HMP: Healthy Michigan Plan
MAGI: Modified adjusted gross income
MDHHS: Michigan Department of Health and Human Services
MTP 2.0: Washington Medicaid Transformation Project 2.0
OUD: Opioid use disorder
PIHP: Prepaid Inpatient Health Plan
PRISM: Promoting Research Initiative In Substance Use and Mental Health
POC: Point-of-care
RI: Recovery Incentives
SAMHSA: Substance Abuse and Mental Health Services Administration
SOR: State Opioid Response grant
StUD: Stimulant use disorder
SUDs: Substance use disorders
TTA: Training and Technical Assistance
TRUST: The Treatment of Users of Stimulants pilot program
UCLA: University of California Los Angeles
WSU: Washington State University
