# Supplementary material for: The path forward for substance use disorder treatment using contingency management under sect. 1115 demonstration waivers
Source: Subst Abuse Treat Prev Policy. 2025 Sep 30;20:37. doi: 10.1186/s13011-025-00666-6 (PMC12486863; doi:10.1186/s13011-025-00666-6)
Supplement: Supplementary file 1 — Supplementary material 1. [file 13011_2025_666_MOESM1_ESM.docx]

## Supplemental Tables and Figures

**Supplemental Table 1:** Links to 1115 Waivers by state as of March 2025

| State | Links to 1115 Waivers^1^ |
| --- | --- |
| California | <https://www.medicaid.gov/medicaid/section-1115-demo/demonstration-and-waiver-list/81046> |
| Washington | <https://www.medicaid.gov/medicaid/section-1115-demo/demonstration-and-waiver-list/83531> |
| Montana | <https://www.medicaid.gov/medicaid/section-1115-demo/demonstration-and-waiver-list/126901> |
| Delaware | <https://www.medicaid.gov/medicaid/section-1115-demo/demonstration-and-waiver-list/81256> |
| Michigan | <https://www.medicaid.gov/medicaid/section-1115-demo/demonstration-and-waiver-list/82141> |
| Rhode Island | <https://www.medicaid.gov/medicaid/section-1115-demo/demonstration-and-waiver-list/83101> |
| West Virginia | <https://www.medicaid.gov/medicaid/section-1115-demo/demonstration-and-waiver-list/83561> |

^1^Active URLs as of March 31st, 2025

## 
